# Group Gratitude: A Taxonomy

**DOI:** 10.1007/s10790-022-09924-3

**Published:** 2023-01-12

**Authors:** Joshua Cockayne, Gideon Salter

**Affiliations:** 1 School of Divinity, University of St Andrews, St Andrews, UK; 2 Department of Psychology, University of Sheffield, Sheffield, UK

## Introduction

There has been an explosion of work on gratitude in the past two decades. Much of this work, led by Robert Emmons and colleagues, has focused on the therapeutic benefits of gratitude, demonstrating the impact of gratitude interventions on psychosocial wellbeing.^1^ In parallel with this growing interest in the practical benefits of encouraging gratitude has been an increase in conceptual work exploring what exactly gratitude is.^2^

However, one frequent omission from conceptual work on gratitude is the focus on gratitude in groups, rather than merely individuals. As Jo-Ann Tsang has recently observed, “the majority of research on gratitude focuses on single recipients of gratitude…The prototypical situation is one benefactor providing a benefit to one recipient.”.^3^ But as Tsang continues: “humans are social creatures. We experience benefits on a group level, as well” leading her to ask, “Do people still experience gratitude, even if the intended recipient is broader than themselves?”.^4^ Tsang is surely right; if we are to understand the nature of gratitude it will not suffice to simply understand how individuals might be said to have the virtue of gratitude or to feel emotions of gratitude.

Consider the following examples:

1. You receive a Christmas present addressed to your whole family, and send a note to thank the gift giver signed “The Smith family”.
2. You receive a thank you gift from a couple after letting them stay with you for a few days.
3. On your last day at work, your employer sends you a letter of thanks and a large cheque thanking you for all your hard work over many years.

Who is expressing gratitude in these examples? You individually, or the whole family? The couple, or each individual? The individual members of your workplace, your boss, or the organisation? There are further questions that arise regarding the emotional experiences involved in such cases. If your brother is not grateful for the joint gift but you are, is your family still grateful for the gift? If you find out that your friend’s spouse didn’t feel grateful for your hospitality, did the couple still express gratitude? If your colleague didn’t contribute towards your leaving gift, is gratitude still being expressed by the *whole* organisation?

These are complex questions. But to begin to answer them, we need some clear ways of distinguishing between different kinds of gratitude phenomena in these contexts. The aim of this paper is to offer a taxonomy of group gratitude that can provide a conceptual tool from which to investigate the group nature of gratitude in both empirical and theoretical projects. The paper proceeds as follows. First, we discuss the ways gratitude has been conceived in the current literature, highlighting its complex and multifaceted nature. Pursuing a strategy of “fractionation”,^5^ different sub-concepts of the broader gratitude concept are identified. These are then linked to areas of psychological and philosophical study that can be drawn upon to contribute to an understanding of the various facets of gratitude at the group level. After outlining the potential insights to be gleaned from these different areas of research, a taxonomy of group gratitude is outlined. The first is *group-context* gratitude, in which the gratitude of an individual is nested in a broader group context, such as a community or a family. Second is *joint* gratitude, in which two or more agents actively coordinate their attention, behaviour and affect in order to be grateful together. Finally, we consider examples of *collective* gratitude, in which organisations or institutions can be said to be grateful, even in the absence of active, co-present coordination, and in cases in which not all of the members of such groups are grateful. Each of these categories has complexities that warrant further investigation, and suggestions are made regarding the ways in which these different kinds of group gratitude might be investigated in future research. While we think that most instances of group gratitude should be considered a species of genuine gratitude, our contention is that the principal contribution of our discussion is not that of introducing a new category of gratitude, but rather, of offering a tool by which to analyze group phenomena that seem to be expressive of gratitude (even if only analogously so).

## Gratitude Concepts and Joint Concepts

As Liz Gulliford and colleagues note, there is far from a unified account of what gratitude is.^6^ Gratitude, they note, has been characterized as an action (e.g., engaging in a behaviour that shows one is grateful), and as an emotion (for instance, “Gratitude is an emotion, the core of which is pleasant feelings about the benefit received.”^7^) Alternatively, within a virtue ethics framework, Gulliford and colleagues suggest, gratitude might be conceived of in episodic terms (i.e., a sequence of behaviours that combine to form an expression of gratitude), or it may be described as a trait (i.e., the property of an individual who consistently displays grateful behaviour).^8^ Gulliford and colleagues thus conclude that the study of gratitude currently resembles a “complicated network of overlapping and criss-crossing concepts”^9^ and suggest that “psychologists slow down their hurry to publish papers on the effects of gratitude to spend more time examining key conceptual issues”.^10^ One might conclude that a unified concept of gratitude might be required to proceed with a definition of group gratitude. However, we argue that the process of articulating a framework for understanding group gratitude might in fact contribute to conceptual discussions relating to the concept (or to concepts) of gratitude.^11^

The approach taken here is one of *fractionation*, whereby “sub-concepts” of the “umbrella concept” are identified and examined, rather than focusing on all facets of gratitude in every study or conceptual discussion.^12^ For example, the actions involved in expressing gratitude can be discussed separately from questions regarding whether gratitude is a distinct emotional category or a mixed emotion. Indeed, as Gulliford et al. note, in “the earliest psychological writings about gratitude, there was no mention of a necessary emotional response. Bertocci and Millard defined gratitude as ‘the willingness to recognize that one has been the beneficiary of someone’s kindness, whether the emotional response is present or not.’”^13^ One can highlight how gratitude interacts with mental health,^14^ or one can discuss how religious people experience a sense of gratitude to God.^15^ The fractionation strategy has proved useful in discussions of other such nebulous concepts as “religion” and “ritual”;^16^ by focusing on specific sub-concepts, conceptual disagreement can be substantive rather than semantic.

Our strategy is therefore to examine specific components of the umbrella concept of gratitude. The aim is not to try and provide a singular definition of gratitude, but instead to focus on sub-concepts that might provide helpful points of contact with other relevant domains of investigation in psychology and philosophy, specifically those that have explored the various joint and collective facets of human experience and behaviour. By drawing upon pre-existing conceptual resources, it is possible to chart a path forward for understanding the various complexities of group gratitude. In each case, we identify a sub-concept that has previously been discussed in the gratitude literature, and link it with a corresponding area of research that has explored joint or collective instances of that sub-concept.

First, gratitude involves *attention*; attending to a source of gratitude and not towards other distractions that minimise gratitude.^17^ This component can be linked to research on *joint attention*;^18^ the ability to coordinate attention to some feature of the world with another and to be aware that this attention is shared.^19^ Joint attention has repeatedly been highlighted as the foundation of mutual awareness of the world,^20^ and plays a key role in the sharing of subjective experiences.^21^ Joint attention thus provides an avenue for understanding the mutuality of sources of gratitude, and joint experiences of grateful feelings.

A second component of gratitude is action. It is generally accepted that gratitude will typically involve some kind of active response.^22^ Thus, research on *joint action* is highly relevant to exploring instances of group gratitude in its various kinds.^23^ Joint action research has sought to articulate what characterises distinctively joint modes of acting, focusing both on the coordination of bodily movements in space and time and the coordination of mental representations to achieve planned outcomes.

A third component of gratitude is the notion of gratitude as a *trait* or *disposition*, a property of those who consistently act in a grateful manner.^24^ This component can be linked to discussions of *group virtue*s,^25^ and how to understanding groups, organisations or communities as having properties such as “welcoming” or “grateful”.

These various domains that will be drawn upon to understand group gratitude agree on a key, fundamental point: understanding group gratitude involves more than a summation of properties of individuals. The *couple* expresses gratitude and not just two people. The *organization* expresses gratitude and not just its employees Understanding group gratitude thus requires more than applying conceptions of individual gratitude to multiple agents; it requires drawing from new conceptual resources that can articulate what marks a situation as a joint or shared. Our aim in discussing these domains is to explore any expression of gratitude that involves two or more people. In a weak sense, this is all we mean by ‘group gratitude’. As we attempt to offer a taxonomy of group gratitude, we show that what is involved in group gratitude will differ significantly in different cases. In the final section we consider whether group gratitude should really count as an instance of gratitude at all.

### Sharing the Experience of Gratitude: Joint Attention and Shared Subjective Experiences

The first sub-concept of gratitude that we focus on is *attention.* Writing on gratitude more generally, Emmons and Stern write the following:

“Gratitude practice is systematically paying attention to what is going right in one’s life… Gratitude practice is intentionally shifting your attention from the negative to the positive…”^26^

A focus on the role of attention in practicing gratitude can be linked to work in developmental psychology and philosophy on *joint attention*,^27^ the ability to coordinate attention to a common target with mutual awareness that this target is shared.^28^ Joint attention, in a strict sense of “joint”, is thus more than two individuals simultaneously attending to a common target, but requires reciprocity and mutuality between co-attenders.^29^ Developmental psychologists have highlighted the crucial role that joint attention plays in social development, as well as human cognition and culture more broadly.^30^ Typically emerging in the latter half of infants’ first year, joint attention is initially achieved through gaze coordination,^31^ touch,^32^ and gestures.^33^ Later in development, language becomes the primary means of coordination joint attention,^34^ and the targets of joint attention are not solely physical objects or stimuli, but can include abstract targets such as mental contents like thoughts and beliefs.^35^

Theoretical accounts have emphasised that in cases of joint attention, there is a characteristic “openness” regarding the attentional target,^36^ as participants actively share and communicate about it.^37^ Joint attention has thus been described as enabling co-attenders to achieve a shared or mutual awareness of features of the world, whether concrete or abstract.^38^ In the case of gratitude, it is thus plausible that just as individual gratitude involves attending to a source of gratitude (e.g. to the positives in one’s life),^39^ so joint attention involves jointly attending to some shared source of gratitude. For example, whilst two partners can individually be grateful for the arrival of their new child, they can also jointly attend to this source of gratitude. To do so *jointly* is plausibly a distinct kind of experience to doing so individually, as we will go on to explore.

The effects of joint attention go beyond rendering the source of gratitude as shared. Joint attention plays a role in achieving a shared experience of subjective inner states.^40^ Various theoretical accounts have highlighted that joint attention plays a key role in rendering subjective emotional experiences as shared. Echterhoff and colleagues argue that by communicating attitudes to a common referent, individuals can achieve a sense of “shared reality”; commonality with others’ inner states about the world.^41^ Focusing more on the ability of bodily expressions to reveal emotional experiences, León and colleagues argue that shared emotions are achieved when individuals engage in joint attention to a common focus and allow their emotional responses to that focus to mutually influence one another.^42^ Through these responsive engagements, participants achieve a sense of commonality between their private subjective experiences. It is important to note that these various account of shared subjective feelings do not deny that there are differences in individually-experienced phenomenology. Rather, they emphasise that, through joint attention, humans have a capacity to create a sense of sharedness about their subjective experiences.

It is also relevant to note that shared experiences have an amplifying emotional effect.^43^ Boothby and colleagues found that pleasant and unpleasant flavour sensations were felt to be more intense when experienced simultaneously with another. These findings support the notion that sharing attention to a stimulus has an amplificatory effect, making that experience have a greater psychological salience.^44^ Thus, it may be the case not only that individuals can experience a sense of shared feelings of gratitude, but that experiencing grateful feelings with others can be a stronger emotional experience.

Individuals can also have a sense of joint experience even without active communication or the physical presence of others.^45^ The work of Shteynberg and colleagues has shown that subtle manipulations of individuals’ beliefs about sharing attention can induce genuine effects on learning,^46^ mood,^47^ and political evaluations.^48^ For example, Shteynberg and colleagues had participants in one condition watch a video of a political speech which was marked “previously recorded” and had a static number indicating previous views. In the other condition, participants (watching the same video) were told they were watching a live feed, with a changing viewer count.^49^ Controlling for extraneous factors, the study found that participants reported the speech as more persuasive in the “live” condition, suggesting that simply creating a sense of shared attention influenced participant judgements.

Given the complex nature of experiences that can fall under the terms “joint” or “shared”, it has recently been argued that shared experiences lie on a scale.^50^ Whilst the strongest kinds of joint experience involve active, communicative, physically present engagements, there are also minimal joint experiences that can occur in the total absence of others.^51^ As we explore in our taxonomy, group gratitude experiences can be similarly diverse, from a couple celebrating together to an individual being aware that others are also grateful for the actions of healthcare workers. Thus, being able to draw upon these conceptual resources will be beneficial for understanding different kinds of group gratitude in our taxonomy.

### Acting Gratefully with Others

The second sub-concept we focus on is *action,* which can be linked to the topic of *joint action*. If we consider examples of groups acting together, whether sports teams, orchestras, or actors, there is a sense in which some group activities go beyond the actions of each individual. As Woodworth articulated:

Two boys, between them, lift and carry a log which neither could move alone.

You cannot speak of either boy as carrying half the log… Nor can you

speak of either boy as half carrying the log… The two boys… achieve a result which is not divisible between the component members of this elementary group.^52^

What then marks a coordinated action as joint? Philosophical accounts tend to broadly agree that the difference is that the latter case involves some kind of “shared intention” or “we-intention”.^53^ In other words, there is a form of activity that is distinctively joint, as in John Searle’s discussion of we-intentions present in joint actions . There has been significant debate regarding how exactly to characterise the “jointness” of joint action,^54^ and these discussions have spawned a large and growing literature on joint action in the psychological sciences.

Psychologists have distinguished *emergent* and *planned* joint actions.^55^*Emergent* joint action refers to those cases in which joint action occur spontaneously and without explicit planning, while *planned* joint actions involve agents agreeing on a goal and working to achieve it. Studies of emergent joint action have typically come from researchers interested in temporal coordination of bodily actions and the human tendency to synchronise bodily movements, even unconsciously.^56^ Investigations of *planned* joint actions focus on higher-level cognitive representations such as intentions, beliefs and commitments,^57^ with a greater focus on the role of language.^58^ In practice, there is typically an interplay between these different kinds of joint action.^59^

Turning to the intersection of joint action and gratitude, it would seem reasonable to suggest that there are cases in which grateful actions are joint actions. If, for example, a couple received a gift from a friend, they could express gratitude individually; one might bake the generous friend a cake, and the other could write the friend a letter. But there can also conceivably be cases in which the grateful response is joint; both individuals take a cake to the friend, or both write a letter together. In such cases, it would appear that the most appropriate way to understand these grateful actions is as jointly grateful actions, which cannot be credited half to one person and half to the other, but wholly to both.

Joint action also provides a useful way of understanding ritual expressions of gratitude. Different religious traditions engage in communal activities such as prayers, ceremonies and fasts that are understood as active expressions of gratitude.^60^ For example, the Christian practice of the Eucharist (literally, thanksgiving) and the Jewish festival of Passover each provide an occasion for a religious community to jointly express gratitude to God through reciting lines of liturgy and the acts of ritual eating and drinking.^61^ Both of these rituals fit more readily under planned coordination than emergent coordination, in that there is a joint goal that can be achieved (expressing gratitude) through a variety of contributory actions that need not involve bodily coordination, though may do so. However, it may also be the case that participating in practices that involve coordinated movements have emergent features, as in the case where spontaneous gratitude emerges from scripted liturgy—the scripted practice of the Eucharist might prompt one to be grateful in a way that is not contained within the confines of the ritual’s script.

An interesting possibility of joint grateful actions is that one can participate without the emotional experience of gratitude. Using our example, John might know that the generous friend had obtained their money through illegal activity, and thus is not grateful for their gift. However, rather than reveal this knowledge, he may choose to jointly act in a grateful manner with Betty. One might argue that both actors must experience grateful emotions for a genuine case of joint grateful action. But for Betty, it seems plausible to say that there would be no difference in her experience. The possibility of a mismatch between the individual level and group level is an issue we return to in our taxonomy.

### Gratitude as a Joint Trait

We move from talking about instances of jointly grateful actions to discussing jointly grateful traits. We might, for instance, plausibly talk of a couple who reliably and consistently express gratitude together over a long period of time, such that we might rightly say: “Jon and Betty are disposed towards expressing gratitude”.^62^ We certainly think it makes sense to talk of virtues in such manner. In fact, some philosophers have thought that two or more agents jointly committing to a way of action, under suitable conditions might rightly be described as exhibiting group level virtue. For instance, Fricker writes that,

If, under conditions of common knowledge, a number of individuals commit to a virtuous moral or epistemic motive, they thereby constitute themselves as the plural subject of that collective motive. … a joint commitment to a virtuous motive is a matter of jointly committing to the virtuous end for the right reason. Note that group members need not possess the motive as individuals. Rather, in jointly committing to it, they each come to possess it qua member of the group… add to this group motive the requisite reliability condition; and voila, we have a collective virtue.^63^

Thus, on Fricker’s account, if Jon and Betty jointly commit to the motives of gratitude (they might, for example agree to prioritise the practicing of gratitude together by reading Emmons’, *Gratitude Works!* together each night) and if they live up to these motives (i.e., if Jon and Betty do regularly express gratitude together in response to gifts), then, it follows for Fricker’s account that Jon and Betty display these virtues jointly, or as a group (i.e., the group ”Jon and Betty, the couple”). Unlike some accounts of group membership we will consider in this paper, Fricker’s account is relatively thin. For Fricker, if Jon and Betty jointly commit to a course of action, they constitute a group. Thus, we might say: Jon and Betty are a grateful couple, even if we might assess their traits as individuals differently. But Fricker’s analysis will struggle to explain other instances of group gratitude, such as whether a university or a corporation is grateful (if indeed we can make sense of these as genuine grateful concepts at all).

### The Limitations of Joint Gratitude Concepts

Finally, it is important to see that there are limitations to these kinds of “joint” concepts. As Stephanie Collins describes, joint action “rises and falls with the specific joint commitment that defines it—for example, a joint commitment to paint the house or go for a walk.”^64^ But not all instances of group action seem to *rise and fall* in quite this way. For example, large organisations appear to persist even when key members leave, and this does not mean that they are unable to continue to perform group actions, or to act virtuously (or indeed, viciously). Consider how a newspaper might be said to display the virtue of courageous journalism. While a team of investigative journalists might display courage through joint actions (such as the exposure of abuse by the *Boston Globe* as depicted in the movie, *Spotlight*), we might also say of a newspaper that it is courageous over a long period of time (i.e., we might say that the *Boston Globe* has consistently acted courageously in pursuit of the truth for the past two decades, even though its editorial team have changed entirely over this period). Joint action accounts do not allow us to say much of the long-term actions or virtues of a group if there are changes in constitution.

Moreover, in large, dispersed groups in which work is delegated, joint action accounts fail to capture a plausible account of the group’s acting. For plausibly, I might contribute to the actions of a group without having any awareness of the aims of the larger group. For example, the *Boston Globe* might recruit a journalist to write an article that involves a long-term covert investigation (i.e., because of its sensitive nature), such that the rest of the staff are not aware of the journalist’s actions and the journalist is unaware of the actions of the group. When the article is eventually published it is still the case that *the Boston Globe* has produced a piece of courageous journalism. But it does not do this in virtue of joint action of any discernible kind. The same would clearly apply to cases of group gratitude. That is, there are plausibly instances of gratitude in which large, dispersed groups might be said to act gratefully. And so, we must look beyond *joint* gratitude if we wish to provide a comprehensive taxonomy of group gratitude.

Consider an example. Suppose a university receives a large financial gift from one of its donors. The university hierarchy meets together to decide what the best response to this donation might be, and after some deliberation, decide to send a letter on behalf of the university, as well as naming one of their faculty buildings after the benefactor. In the letter, the University Principal writes the following words: “On behalf of the university I would like to express my deep gratitude for your donation; this gift will benefit many students for many years to come”. Who is the benefiter in this instance? Not the principal, or at least not primarily. Not a group of students who are jointly grateful for the gift, either. Rather, we suggest, the *university* is the benefiter of the donation and the Principal’s letter express gratitude on behalf of the university as a group.

How should we make sense of such claims? According to some philosophers, collectives can be thought of as agents, capable of performing actions (such as a corporation spilling oil in the North Sea), and even as moral agents capable of displaying virtues and vices (such as a corporation having the vice of greed). In order to explain collective gratitude, we need to provide an analysis of what it is for that group to act; that is, to explain who is authorized to act on behalf of the group and who is authorized to participate as a member of the group.

This need not commit us to thinking that there are mysterious entities called, “groups” which exist beyond individual agents, to which we can ascribe virtues. Rather, the thought goes, certain organisational structures and decision-making procedures allow groups of individuals to act in such a way that they can be said to be the agents of certain actions. Collins suggests that collectives are groups which are “constituted by agents that are united under a rationally operated group-level decision-making procedure that can attend to moral considerations.”^65^ For example, the British government is constituted by the ministers of the cabinet, who, through a series of group-decision making procedure, deliberate on the “best” course of action for the country. Collectives, unlike groups involved only in joint action, have “an identity that can survive changes of membership,”^66^ such as “a nation, a university, or a purposive organisation.”^67^

While joint intentionality clearly plays a role in such procedures, for the reasons suggested above, it cannot be said to give a complete account of group action. The reason for this is that members of groups don’t all contribute to each action a group performs. Typically, in collectives there are authorising members (i.e., those who permit others to act on their behalf, like fee paying members of a trade union) and active members (i.e., those who are authorized to act on behalf of the group, say by lobbying an employer on behalf of a trade union). As List and Pettit argue, in a collective:

…two types of members are typically present and often overlap. In a participatory group like a voluntary association, members have the same status within the group agent; they equally authorize the group agent and take roughly equal parts in acting on its behalf. In a hierarchical organization, such as a commercial corporation or a church, there may be differences in the members’ roles, for example through holding different offices or through belonging to subgroups with different tasks.^68^

In extreme cases, such as a dictatorship or autocracy, there may be one active member who acts on behalf of the group, and many more authorising members who do not have the authority to act on behalf of this group. However, typically in cases like organisations like universities, the authority is more dispersed. Return to our example of the university’s letter. In an organisation such as a university some are authorised to speak on behalf of the collective- the principal of a university is elected or appointed to have such authority, but there are likely many layers of authority beneath this too. Committees are appointed to make decisions on behalf of the university in issues of policy and governance. But then there are many members of the university who are unable to contribute to its agency directly; an undergraduate student does not have the authority to write a letter of thanks *on behalf* of the university. However, by becoming a member of the university she authorises (perhaps implicitly) the Principal to express gratitude on her behalf.

Thus, it may the case that not all members of a group are involved in expressing gratitude directly. We can see this by considering a phenomenon some social psychologists have called “social loafing”, in which the group carries members who do not or cannot contribute to joint actions.^69^ This might be a negative phenomenon where the individual is unwilling (such as in the case of a grumpy person in a grateful group), but it might also be a positive phenomenon, such as in cases where the individual is unable*.* For example, a person may not fully participate in the gratitude of the group because they are having a difficult time in life, or because of certain disabilities preclude them from participating, or limit their participation in joint actions.

Whether full participation is influenced by being unwilling or unable, those that are not feeling or expressing gratitude as an individual have a means to express gratitude by virtue of being nested within a grateful joint action and/or a grateful collective. In the case of joint action, even minimal participation (e.g., being present for a ritual expressing thanks, but not being willing or able to join in the actions) constitutes a form of participation, and is thus a means of expressing gratitude. In cases where an individual does not or cannot contribute to joint grateful actions (e.g., choosing not to join, or being physically unable to join a joint action), they can still be part of a grateful collective as a member, as long as they align with the membership conditions for that collective. For example, an elderly congregant who only watches a church’s services online because they fear contracting COVID is still a member of that community. To the extent that the unwilling cases are distinct from the unable cases, we would suggest that collective membership conditions are typically more lenient towards unable cases. There is also a question of the extent to which joint action participation relates to membership of the collective; it seems reasonable that an individual needs to be willing to participate in at least some of the joint actions of a group to be considered a member of the collective, but precise boundaries are difficult to draw and likely to vary between different collectives (e.g., a business would quickly jettison an employee who rarely participated in the joint actions of the company, but an amateur sports club might allow a member who rarely attends practices and matches).^70^

Moving from episodic gratitude to gratitude as a trait should be relatively straightforward. If a collective is able to consistently perform acts of gratitude through rationally operated group-decision-making procedure, then we have no reason not to think that a group like a university cannot be said to display the virtue of gratitude. Just as the *Boston Globe* might be said to display courage, the university can be said to display virtues that might not be represented by each of its members. Byerly and Byerly provide one such account. They argue that, “A collective C has a virtue V to the extent that the members of C are disposed, qua members of C, to behave in ways characteristic of V under appropriate circumstances.”^71^ For instance, they suggest, “a collective C has the virtue of intellectual humility when it is disposed to display striking or unusual unconcern to be well-regarded by others.”^72^

Thus, to apply such an account to gratitude would be to say that a collective, say, a university, is organised such that its authorised members consistently act in a way that exhibits grateful behaviour. So, for example, the university might consistently write letters of thanks to donors, or acknowledge long-standing service in its faculty newsletter, and so on. These discussions provide a means of thinking about how groups of various types might be considered grateful, and not solely in situations in which multiple agents are simultaneously grateful. Rather, if a group has the trait of being grateful, the group’s grateful nature does not depend on the gratitude of any particular individual.

## A Taxonomy of Group Gratitude

Having highlighted the various research that can contribute to an account of group gratitude, we now present a taxonomy of group gratitude. Table 1 displays the different levels of the taxonomy and their distinguishing features.

**Table 1:** Levels of group gratitude and their distinguishing features

Type of gratitude	Who is the subject expressing gratitude?	Role of awareness	Is joint action present?	To what extent are grateful feelings involved?
Individual gratitude	Individual (e.g., Jon)	Individual awareness of source of gratitude, where that individual is the sole beneficiary	No	The individual experiences grateful feelings
Group-context gratitude	Individual (e.g., Jon)	Some awareness that others are grateful to the same source, whether simultaneously (online) or not (offline)	No	The individual experiences grateful feelings
Joint gratitude	Two or more individuals (e.g., Jon and Betty)	Occurrent shared awareness of the source of gratitude amongst all participants	Yes	Joint grateful feelings may be present, but not in all cases
Collective gratitude	Collective (e.g., The University)	Collective *identifies* source of gratitude through the joint decision-making procedures of its members. The collective itself is not *aware*	Sometimes	Not involved at the collective level, though there may be joint or individual grateful feelings within the collective

### Individual Gratitude and Group-context Gratitude

The first levels are *individual gratitude* and *group-context* gratitude. These levels are similar, in that there is a single grateful agent in each case. However, the difference that marks cases of group-context gratitude, gratitude is experienced or expressed in a group setting but the grateful agent is an individual, rather than a group. Consider Tsang’s example. She imagines a community stricken by natural disaster receiving money to fund the rebuilding of the whole town.^73^ Such a case, Tsang thinks, is different to a case in which an individual’s house is rebuilt after a natural disaster. For in the group case, the individual benefactor—the recipient of a new house, is nested in a broader community—the town. While the benefactor of the benefit in such cases is a group, gratitude is still described as something primarily attributable to individuals. All that the group provides is the context for such gratitude.^74^

In such cases, one is not experiencing gratitude jointly with others, nor is a group entity said to express gratitude in any meaningful sense; it may occur in total absence of interaction with or presence of others. Thus, while we include contextual group gratitude in our taxonomy, it is more straightforward to analyse within existing gratitude frameworks, since the benefactor is not a group or a collective of individuals, but an individual who situates their experience within a broader context. Furthermore, we can further divide contextual group gratitude between “offline” and “online” cases. Offline contextual group gratitude requires a minimal awareness that one shares a reason for gratitude with others, but with no sense that others are doing so at the same particular moment. A relevant example from the time of writing is “vaccine gratitude”; one can be grateful that they have received a vaccination, knowing that others across the country are also grateful, despite these acts of gratitude not necessarily occurring simultaneously. The act is not entirely individual, as one knows one’s gratitude is likely to be occurring in the same broad timeframe as others. However, it is “offline” because it is not clearly occurring simultaneously with others. In contrast, online contextual group gratitude requires awareness that one’s individual gratitude is occurring simultaneously with others who are also being grateful for the same reason or reasons. For example, the “clap for carers” movement in the UK had people across the country expressing gratitude (by clapping, banging pans or playing music) at a particular time each week during the national lockdown brought on by COVID-19. Even those who did not (or could not) actively participate, or lived away from others (i.e., could not express gratitude *jointly*), could take a moment to be grateful for the nation’s carers, aware that many others were doing so at that specific moment. But in most cases, it did not rise to the level of joint gratitude, since there was no joint attention or joint intentionality present in the expression of gratitude.

Psychological research on shared experiences can provide the evidential basis for these claims about group-context gratitude. The sense of shared experience is generated by beliefs about jointness, even in the absence of any direct interaction with others.^75^ As the work of Shteynberg and colleagues has shown,^76^ humans are remarkably sensitive to even minimal cues to an experience being shared, and thus it is plausible that demonstrable effects on gratitude might be found in cases where the gratitude of individuals is nested within a group context.

In Tsang’s study, we already have example of empirical work that focuses on how individuals understand cases of gratitude that are nested within a group context. Future empirical can build on this work by exploring the differing emotional, cognitive and behavioural effects of online and offline contextual group gratitude. We might expect (thanks to Shteynberg and colleagues) that experiencing offline and online contextual group gratitude have differing effects; if one practices gratitude simultaneously with others, even others who are not physically present, it may produce stronger feelings of gratitude or greater likelihood of grateful action than just the knowledge that others share the same reasons to be grateful. For example, participants in a study could be sorted into two groups. In the first group, participants are given a scheduled time each day when they are to complete a gratitude diary, and are informed that they are doing so simultaneously with other participants. In the other condition, participants are told they are assigned a random time in the day to complete a daily gratitude diary, with other participants also assigned different random times. However, all participants in both conditions would complete the diary at the same time of day, to avoid time of day being a confound. Thus, the difference between conditions would be the awareness that others are completing the diary at the same time. It is possible that participants in the first (aware of simultaneity) group are both more likely to experience the benefits of the diary, as well as feeling more committed to the activity and thus completing it more consistently.

### Joint Gratitude

The next level is *joint gratitude*. Joint gratitude involves 1) jointly attending to the source of gratitude, 2) co-attenders actively signalling their grateful attitude to that source (even if this attitude is not always identical in all participants), and 3) jointly responding with some kind of grateful action. Joint gratitude involves shared awareness of the source of gratitude and individuals’ response to it. Similarly to individual cases, a group of jointly grateful individuals can be assigned the trait of gratitude if they persistently engage in grateful behaviours. However, it is important to note that joint gratitude need not involve *identical* levels of experience and involvement amongst all participants. Not only will participants have a distinct subjective experience,^77^ but they do not necessarily all contribute equally to jointly grateful actions.^78^

There are a variety of ways in which joint gratitude could be investigated. First, building on previous work on affective responses in the joint context,^79^ it might be expected that jointly grateful experiences typically induce stronger emotional responses than individual experiences. This could be assessed using a variety of measures, from participant reports^80^ to physiological responses (e.g. increased heart rate and synchrony^81^). Similarly, jointly experiencing gratitude may induce stronger effects on social bonding than simultaneously experiencing gratitude, even if the source of gratitude is common between individuals.^82^ Diary methodologies could also investigate the impact of joint gratitude, for example by asking couples to keep a gratitude diary either jointly (i.e., completing it together about shared sources of gratitude) or individually (e.g., completing a diary separately about shared sources of gratitude).

It is also possible to make predictions regarding how joint gratitude is distinct from group-context gratitude. Firstly, the gratitude of each agent at the joint gratitude level is public or open in a way not true of group-context cases. This enables potential joint grateful actions to proceed in a manner not possible in cases of contextual group gratitude, where the source of gratitude is not necessarily out in the open. This sharedness also creates a sense of commitment to acting in a grateful manner that is not necessarily the case at the group-context level.^83^ Jointly grateful agents are normatively committed to this fact, and cannot deny they were grateful without significant social cost. Practically, this creates obligations to act,^84^ whether communicating one’s gratitude or reciprocating in some manner. In cases of group-context gratitude, agents do not face the same normative expectations, and thus may be less likely to respond in a grateful manner. One way to investigate whether this is the case is to create a situation in which participants have a choice whether or not to express gratitude, and to examine whether participants are more likely to engage in personally costly actions (such as giving up time or resources) to express gratitude in cases of joint gratitude versus individual or group-context gratitude.

Finally, whilst the definition we have offered focuses on the coordination of attitudes to the shared source of gratitude, it is possible that there are cases in which individuals participate in joint gratitude without the concomitant grateful feelings. Whilst it could be argued that this is not a genuine case of joint gratitude, it may be conceptually useful to examine cases in which individuals communicate they are jointly grateful, whilst privately not experiencing grateful emotions. It is possible that such situations can have positive and negative consequences. Positively, such situations may be a route into grateful feelings; an individual who is not feeling grateful engages in jointly grateful practices, and subsequently is able to attend to sources of gratitude and thus start to feel grateful. On the negative side, feeling a pressure to engage in joint gratitude without grateful feelings may increase individuals’ sense of isolation from others.

Here, we might find it helpful to distinguish between a kind of ‘decisional gratitude’ and an ‘emotional gratitude’. In a related discussion of the concept of forgiveness, Everett L. Worthington, Jr. argues that ‘decisional forgiveness’ (i.e., a wilful act to forgive someone who has wronged you) and ‘emotional forgiveness’ (i.e., the feeling of no longer holding wrongdoing against a transgressor) can come apart. Worthington writes, ‘People could decide to forgive and not experience emotional forgiveness. They also could experience sudden compassion for a transgressor… and realize that unforgiveness had disappeared even though no decision had been made to forgive’.^85^ It is not always the case, Worthington thinks, that emotional forgiveness always precedes decisional forgiveness or vice versa. While it is typically the case that decisional forgiveness leads to emotional forgiveness, this might not always be the case; one’s affective response to a perpetrator might change and lead to a decision to forgive. There may be cases of joint gratitude that are expressions of decisional gratitude, but which lack the emotional component. Whether these count as *genuine* instances of gratitude is really a terminological issue. Nevertheless, the phenomenon of expressing gratitude jointly (with or without grateful emotions) still seems worthy of further discussion independently of this terminological issue.

To summarise, a number of researchers have stressed that understanding different senses of “jointness” or “sharedness” is a complex issue, with a variety of senses and levels.^86^ We have only provided a broad introduction to the notion of joint gratitude, and thus further conceptual work can enrich the various aspects of this concept and explore its potentially varied and diverse effects on cognition, emotion and behaviour.

### Collective Gratitude

The final level of the taxonomy is *collective gratitude*. Collective gratitude occurs when organisations or social groups are organised such that they can act gratefully in response to benefits identified at the collective level. Note that unlike the other kinds of group gratitude in the taxonomy, collective gratitude is not dependent on joint attention, and we assume that there is no collective-level phenomenology. The individuals on whom the collective actions depend on may attend to group-level benefits, but the collective as the subject of gratitude can only *identify* benefits through decision-making procedure (such as voting, or group hierarchy). We also note that the term “collective gratitude” has previously been used in the literature (e.g., Fehr et al., 2017), but in reference to organisations that contain many grateful individuals, an approach that differs to ours here.

We concede that many psychologists will struggle to make sense of collective gratitude as an instance of genuine gratitude if they insist that emotions are a core component of gratitude. It is difficult to make sense of the notion of collective emotion or feeling, even if grateful organisations may foster grateful feelings in their members. Nevertheless, the phenomena of collectives expressing gratitude still seems worthy of exploration in a number of contexts. Moreover, the fact that we cannot talk about emotions does not rule out the possibility of talking about well-formed and badly-formed instances of collective gratitude. Consider an example offered to us by an anonymous reviewer:

Suppose that a group A is merely strategically good at returning someone’s or some group’s favor with a view to its future reputation, while a group B performs similar action motivated by its members’ genuine feeling of gratitude toward the benefactor. The concept of group gratitude should be able to tell the difference between these two groups.

While in cases of individual gratitude we might seek to distinguish these cases by referring to emotional states, clearly this option is not available in the context of collective gratitude. But this doesn’t mean we cannot distinguish between these two cases at all. Take a parallel example of so-called “greenwashing”, where corporations appear to offer environmentally friendly products in order to attract environmentally conscious consumers, even though the company shows a disregard for providing evidence of how sustainable their products really are. Many activists are deeply troubled by these practices, and rightly so. But this isn’t because we want companies to have *emotions* concerning the planet, but rather because we want companies to act for the right *reasons.* We want companies who are committed to the end goal of sustainability, rather than the end goal of profit. Similarly with grateful institutions, what might distinguish genuine gratitude is the reasons for the grateful action. Is the board committed to expressing thanks to its benefactors, or does it merely wish to look good on social media? We can make these distinctions without invoking any emotional states.

The analysis of collective gratitude we have given does not specify what the threshold is for being a case of genuine collective gratitude. However, as a next step, it would be beneficial to clearly articulate the conditions under which a group or organisation can be said to be more or less grateful. If this can be achieved, organisations can assess the extent to which they have achieved this goal or can pursue measures that facilitate this end. However, given that collectives have no shared consciousness or phenomenology, and thereby no feelings of gratitude, it may be the case that there are limitations on the kinds of questions that can be asked at the collective level versus the group-context and joint levels. The focus would primarily be on the way in which collectives make decisions, and the reasons for these decisions.

However, as the quotation above suggests, it is important to consider how individuals- and individual emotional responses- contribute to and are influenced by being a part of collectives that are categorised as being more or less grateful. Some previous work has discussed how to promote a “culture” or “climate” of gratitude in an organisation, suggesting that it emerges as a result of many individual grateful experiences.^87^ Here, we suggest that gratitude in collectives may be more complex, allowing for a mismatch between the experiences of individuals and the categorisation of organisations. An organisation may express gratitude despite a lack of gratitude from its individual members, such as when the organisation expresses gratitude for the purchase of a new building, but many members are resentful that they subsequently need to relocate.

In our framework, we have avoided assuming a straightforward correlation between collective gratitude and individual grateful experiences within that collective. This is a matter for further empirical exploration. Empirical studies could assess the emotional responses of individuals in different collectives that are categorised as being grateful or ungrateful according to their collective-level decisions and reasoning processes, to examine the relation between individual and collective gratitude. Additionally, the effect of joining a grateful collective could be examined. It is plausible that an individual’s gratitude might be facilitated by joining a grateful group, rather than solely or primarily via individual practices.

## Conclusion

This paper has argued that group gratitude is an umbrella concept worth investigating. By fractionating gratitude into its various sub-concepts,^88^ we forged links with different domains of research that can contribute to an understanding of group gratitude. On the basis of the insights gleaned from a range of research in psychology and philosophy, we have proposed a taxonomy of three kinds of group gratitude: group-context, joint, and collective. These kinds of gratitude can be distinguished by asking who the subject of gratitude is, as well as analysing the role of attention, action, and grateful feelings involved in each (see Table 1). However, we recognise that many cases will be difficult to map neatly onto this taxonomy. For instance, in cases of collective action it may be that all three kinds of group gratitude are occurring simultaneously—an organisation which displays collective-level gratitude may do so through the joint actions of many individuals who are also experiencing gratitude individually. While a taxonomy can help pick out important features of these cases, future work must remain sensitive to the fact that the reality of group gratitude will be complex in many cases. Regardless, paying greater attention to different kinds of group gratitude has the potential to enrich the study of gratitude by enhancing conceptual discussions and by providing directions for future research.

## Footnotes

1 See Robert A. Emmons, *Thanks!: How the new science of gratitude can make you happier*. (Boston, MA: Houghton Mifflin Harcourt, 2007); Emmons & Crumpler, C. A. “Gratitude as a human strength: Appraising the evidence.” *Journal of Social and Clinical Psychology*, *19*(1), 56-69, (2000); Emmons & Stern, R. “Gratitude as a psychotherapeutic intervention.” *Journal of Clinical Psychology*, *69*(8), 846-855. (2013); Froh, J. J., Sefick, W. J., & Emmons, R. A. “Counting blessings in early adolescents: An experimental study of gratitude and subjective well-being.” *Journal of School Psychology*, *46*(2), 213-233 (2008).

2 Liz Gulliford, Morgan, B., & Kristjánsson, K. “Recent work on the concept of gratitude in philosophy and psychology.” *Journal of Value Inquiry* 47, 285-317 (2013); Michael E. McCullough,, Kilpatrick, S. D., Emmons, R. A., & Larson, D. B. “Is gratitude a moral affect?” *Psychological Bulletin*, *127*(2), 249-266. (2001).

3 Jo-Anne Tsang, J. A. “(Un) special Favors: Gratitude for Group-Based Benefits.” *The Journal of Positive Psychology*, *16*(1), (2021), p. 27.

4 Ibid., p.27.

5 Boyer, P., & Bergstrom, B “Evolutionary perspectives on religion.” *Annual Review of Anthropology*, *37*, 111-130. (2008); Whitehouse, H., & Lanman, J. A. “The ties that bind us: Ritual, fusion, and identification.” *Current Anthropology*, *55*(6), 674-695, (2014).

6 See Gulliford et al., “Recent work”.

7 Emmons, and Michael E. McCullough (eds.), *The Psychology of Gratitude,* (Oxford: Oxford University Press, 2004), p. 5.

8 See Gulliford et al., op. cit.

9 Ibid., p. 313.

10 Ibid., p. 317.

11 This is not to say that all instances that fit under our umbrella concept, group gratitude, *necessarily* meet the threshold to count as well-formed instances of gratitude. Rather, even if group gratitude turns out to be a related concept to individual gratitude, it is still able to inform future conversations about what gratitude *is.*

12 See Boyer & Bergstrom, op. cit.; Whitehouse & Lanman, op. cit.

13 Gulliford et al., op. cit., p. 294; citing P. A. Bertocci and R. M. Millard, Personality and the Good: Psychological and Ethical Perspectives (New York: David McKay, 1963.

14 See Emmons & Stern, op. cit.

15 See D.H. Rosmarin, Pirutinsky, S., Cohen, A.B., Galler, Y., & Krumrei, E.J. “Grateful to God or just plain grateful? A comparison of religious and general gratitude.” *The Journal of Positive Psychology*, 6(5), 389-396. (2011).

16 See Boyer & Bergstrom, op. cit.; Whitehouse & Lanman, op. cit.; Rohan Kapitány, Kavanagh, C., & Whitehouse, H.. “Ritual morphospace revisited: The form, function and factor structure of ritual practice.” *Philosophical Transactions of the Royal Society B*, *375*(1805), (2020)

17 See Emmons & Stern, op. cit.

18 N. Eilan, C. Hoerl, T. McCormack, & J. Roessler, (Eds.). *Joint attention: Communication and other minds: Issues in philosophy and psychology*. (Oxford: Oxford University Press, 2005); Moore, C. and Dunham, P. J. (Eds.). *Joint attention: Its origins and role in development*. (London: Psychology Press, 2014); Scaife, M., and Bruner, J. S. “The capacity for joint visual attention in the infant.” *Nature*, *253*(5489), 265-266. (1975); Seemann, A. (Ed.). *Joint attention: New developments in psychology, philosophy of mind, and social neuroscience*. (Cambridge, MA: MIT Press, 2011).

19 See Michael Tomasello, M. “Joint attention as social cognition.” *Joint attention: Its origins and role in development*, in Moore and Dunham op. cit., pp.103-130.

20 See B. Siposova, & M. Carpenter, M. “A new look at joint attention and common knowledge.” *Cognition*, *189*, 260-274, (2019).

21 See C.D. Hardin, & E.T. Higgins, E. T. “Shared reality: How social verification makes the subjective objective” in R. M. Sorrentino & E. T. Higgins (Eds.), *Handbook of motivation and cognition, Vol. 3. The interpersonal context* (New York: The Guilford Press, 1996), pp. 28–84; G. Echterhoff, E.T. Higgins, & J.M. Levine, J. M. “Shared reality: Experiencing commonality with others' inner states about the world.” *Perspectives on Psychological Science*, *4*(5), 496-521. (2009).

22 Gulliford et al., op. cit.

23 See G. Knoblich, S. Butterfill, & N. Sebanz, “Psychological research on joint action: Theory and data.” *Psychology of Learning and Motivation*, *54*, 59-101. (2011); N. Sebanz, H. Bekkering, H., & G. Knoblich, G. “Joint action: Bodies and minds moving together.” *Trends in Cognitive Sciences*, *10*(2), 70-76. (2006)

24 See Michael E. McCullough, Robert A. Emmons, & Jo-Anne Tsang, “The grateful disposition: A conceptual and empirical topography.” *Journal of Personality and Social Psychology*, *82*(1), 112-127. (2002).

25 Ryan T. Byerly, and M. Byerly, M. “Collective virtue.” *The Journal of Value Inquiry*, *50*(1), 33-50. (2016).

26 Emmons and Stern, op. cit., p. 853.

27 Eilan et al., op. cit. ; Moore & Dunham, op. cit. ; Scaife & Bruner, op. cit.; Seemann, op. cit.

28 Tomasello, op. cit.

29 See Malinda Carpenter, & K Liebal, “Joint attention, communication, and knowing together in infancy.” In A. Seemann (Ed.), op. cit.; Dan Zahavi, and P. Rochat, “Empathy ≠ sharing: Perspectives from phenomenology and developmental psychology.” *Consciousness and Cognition*, *36*, 543-553. (2015)

30 Michael Tomasello, *Becoming human: A theory of ontogeny*. (Cambridge, MA: Belknap Press, 2019)

31 See Scaife & Bruner, op. cit.; R. Bakeman, & L.B. Adamson, L. B. “Coordinating attention to people and objects in mother-infant and peer-infant interaction.” *Child Development*, 1278-1289. (1984).

32 See M. Botero, “Tactless scientists: Ignoring touch in the study of joint attention.” *Philosophical Psychology*, *29*(8), 1200-1214. (2016)

33 See Elizabeth Bates, *The emergence of symbols: Cognition and communication in infancy*. (New York: Academic Press,1979); U. Liszkowski, P. Brown, P, T. Callaghan, A. Takada, & C. De Vos, “A prelinguistic gestural universal of human communication.” *Cognitive Science*, *36*(4), 698-713. (2012).

34 See J.S. Bruner, J. S. “The ontogenesis of speech acts.” *Journal of child language*, *2*(1), 1-19. (1975).

35 See C. O’Madagain, & M. Tomasello, “Joint attention to mental content and the social origin of reasoning.” *Synthese*, *198*(5), 4057-4078. (2021).

36 See J. Campbell, “Joint attention and common knowledge.” In J. Heal, C. Hoerl, T. McCormack, and J. Roessler (Eds.), *Joint Attention: Communication and Other Minds*, 298–324. (Oxford: Oxford University Press, 2005); C. Peacocke, “Joint Attention: Its nature, reflectivity, and connection to common knowledge.” In Heal et al., op. cit.

37 See Carpenter & Liebal, op. cit.

38 See Siposova & Carpenter, op. cit.

39 See Emmons & Stern, op. cit.

40 See Hardin & Higgins, op. cit.; G. Echterhoff, E.T. Higgins, & J.M. Levine, “Shared reality: Experiencing commonality with others' inner states about the world.” *Perspectives on Psychological Science*, *4*(5), 496-521. (2009); F. León, T. Szanto, & D. Zahavi, D. “Emotional sharing and the extended mind.” *Synthese*, *196*(12), 4847-4867. (2019); Zahavi & Rochat, op. cit.

41 Echterhoff et al., op. cit.

42 León et al., op. cit. See also P. Hobson & J. Hobson, J. “Joint attention or joint engagement? Insights from autism.” In A. Seeman (Ed.) op. cit., pp.115-136.; Zahavi & Rochat, op. cit.

43 See G. Shteynberg, G. “Shared attention.” *Perspectives on Psychological Science*, *10*(5), 579-590. (2015).

44 See E.J. Boothby, M.S. Clark, & J.A. Bargh, “Shared experiences are amplified.” *Psychological science*, *25*(12), 2209-2216. (2014).

45 See Joshua Cockayne and Gideon Salter, “Praying Together: Corporate Prayer and Shared Situations.”. *Zygon,* *54*(3), pp.702-730 (2019); Shteynberg, op. cit.; Siposova & Carpenter, op. cit.

46 See G. Shteynberg, & E.P. Apfelbaum, “The power of shared experience: Simultaneous observation with similar others facilitates social learning.” *Social Psychological and Personality Science*, *4*(6), 738-744. (2013).

47 See G. Shteynberg, G., J.B. Hirsh, E.P. Apfelbaum, J.T. Larsen, A.D. Galinsky, & N.J. Roese, “Feeling more together: Group attention intensifies emotion.” *Emotion*, *14*(6), 1102-1114. (2014).

48 See G. Shteynberg, J.M. Bramlett, E.H. Fles, & J. Cameron, J. “The broadcast of shared attention and its impact on political persuasion.” *Journal of Personality and Social Psychology*, *111*(5), 665. (2016).

49 Shteynberg et al., op. cit.

50 See Siposova & Carpenter, op. cit.

51 See G. Shteynberg, “A collective perspective: Shared attention and the mind.” *Current Opinion in Psychology*, *23*, 93-97.

52 R.S. Woodworth, “Individual and group behaviour.” *The American Journal of Sociology, 44*(6), 823–828 (1939), quoted in G. Knoblich, S. Butterfill, & N. Sebanz, N. “Psychological research on joint action: Theory and data.” *Psychology of Learning and Motivation*, *54*, 59-101. (2011), p. 823

53 See John Searle, “Collective Intentions and Actions.” In *Intentions in Communication,* P. Cohen, J. Morgan, and M. Pollack (Eds.) (Cambridge, MA: MIT Press, 1990), pp.401–415.

54 See Knoblich et al. op. cit.; Sebanz, op. cit.

55 See Knoblich, et al., op. cit.

56 See M.J. Richardson, K.L Marsh, R.W. Isenhower, J.R. Goodman, & R.C. Schmidt, “Rocking together: Dynamics of intentional and unintentional interpersonal coordination.” *Human Movement Science*, *26*(6), 867-891. (2007); R.C. Schmidt, R. C., & M.J. Richardson, M. J. “Dynamics of interpersonal coordination.” In A. Fuchs & V. K. Jirsa (Eds.), *Coordination: Neural, behavioral and social dynamics*, (Berlin: Springer, 2008) pp. 281-308.

57 M. Gräfenhain, M., T. Behne, M. Carpenter, & M. Tomasello, “Young children’s understanding of joint commitments.” *Developmental Psychology*, *45*(5), 1430-1443. (2009); N. Sebanz, G. Knoblich, & W. Prinz, “Representing others' actions: just like one's own?.” *Cognition*, *88*(3), 11-21. (2003).

58 See H.H. Clark, *Using language*. (Cambridge: Cambridge University Press, 1996).

59 Knoblich, et al., op. cit.

60 See Emmons & Crumpler, op. cit.; McCullough et al., op. cit.

61 Joshua Cockayne, and Gideon Salter. "Feasts of Memory: Collective Remembering, Liturgical Time Travel and the Actualisation of the Past." *Modern Theology* 37.2 (2021): 275-295.

62 McCullough et al., op. cit.

63 Miranda Fricker, Miranda. “Can there be Institutional Virtues?” in T. Szabo Gendler & J. Hawthorne (Eds.), *Oxford Studies in Epistemology 3,* 235-252. (Oxford: Oxford University Press, 2010), pp. 241-242.

64 Stephanie Collins, *Group duties: Their existence and their implications for individuals*. (Oxford: Oxford University Press, 2019), p. 56.

65 Ibid., p.12.

66 Christian List, & Philip Pettit, *Group agency: The possibility, design, and status of corporate agents*. (Oxford: Oxford University Press, 2011), p.31.

67 Ibid.

68 Ibid., p.36.

69 See R.C., Liden, S.J. Wayne, R.A. Jaworski, and N. Bennett, “Social loafing: A field investigation.” *Journal of Management*, *30*(2), 285-304. (2004); S.J. Karau, S.J. and A.J. Wilhau, “Social loafing and motivation gains in groups: An integrative review.” *Individual Motivation within Groups*, *15*, 3-51 (2020); S.J. Karau, & K.D. Williams, K. D. Social loafing: A meta-analytic review and theoretical integration. *Journal of Personality and Social Psychology*, *65*(4), 681-706. (1993).

70 With thanks to an anonymous reviewer for their suggestions regarding this paragraph.

71 Byerly and Byerly, op. cit., p.43.

72 Ibid., p.48.

73 See Tsang, op. cit.

74 This is not to say that Tsang discounts the possibility of other forms of group gratitude. Only that individual response to contextual group gratitude was the focus of Tsang’s study. With thanks to Jo-Ann Tsang for this clarification.

75 See Shteynberg, op. cit.; Siposova & Carpenter, op. cit.

76 See Shteynberg et. al., op. cit.

77 See León et al., op. cit.

78 See Liden et al., op. cit.; Karau & Wilhau, op. cit.

79 For example, see Boothby et al., op. cit.; P. Mitkidis, P., J.J. McGraw, A. Roepstorff, & S. Wallot, S. “Building trust: Heart rate synchrony and arousal during joint action increased by public goods game.” *Physiology & Behavior*, *149*, 101-106. (2015); Shteynberg, op. cit.

80 See Boothby et al., op. cit.

81 See Mitkidis et al., op. cit.

82 See W. Wolf, J. Launay, & R. IDunbar, R. I. “Joint attention, shared goals, and social bonding.” *British Journal of Psychology*, *107*(2), 322-337. (2016)

83 See B. Siposova, M. Tomasello, & M. Carpenter, M. “Communicative eye contact signals a commitment to cooperate for young children.” *Cognition*, *179*, 192-201. (2018).

84 See M. Tomasello, M. “The moral psychology of obligation.” *Behavioral and Brain Sciences*, *43*. e56: 1–58. (2020).

85 E.J. Worthington, J. *Forgiveness and reconciliation: Theory and application*. (Milton Keynes: Routledge, 2013), p.25

86 See Echterhoff et al., op. cit.; Siposova & Carpenter, op. cit.; Zahavi & Rochat, op. cit.

87 See A. Di Fabio, L. Palazzeschi, and O. Bucci, “Gratitude in organizations: A contribution for healthy organizational contexts.” *Frontiers in Psychology, 8*, 1-6. (2017); R. Fehr, A. Fulmer, E. Awtrey, and J.A. Miller, “The grateful workplace: A multilevel model of gratitude in organizations.” *Academy of Management Review*, *42*(2), 361-381. (2017).

88 See Boyer, op. cit.; Gulliford et al., op. cit.; Whitehouse & Lanman, op. cit.

